# Personality disorder not otherwise specified heterogeneity and its implication in psychiatric residential treatment

**DOI:** 10.1192/j.eurpsy.2021.626

**Published:** 2021-08-13

**Authors:** L. Bailo, P. Vimercati, M. Caslini, S. Damiano, E. Pellegrini

**Affiliations:** Comunità Psichiatrica Villa Ratti, Società cooperativa sociale IL VOLO-Onlus, Monticello Brianza, Italy

**Keywords:** PDNOS, Therapeutic Community, Diagnostic agreement

## Abstract

**Introduction:**

Villa Ratti is a therapeutic community dedicated to the treatment of Personality Disorder with a particular focus on Borderline Personality Disorder (BPD), but this diagnosis may manifest in very different clinical conditions (Bayer & Parker, 2017; Scott, 2017).

**Objectives:**

Since the second most common diagnosis we encounter from referring psychiatrists is Personality Disorder Not Otherwise Specified (PDNOS) (26,4%) and this diagnosis serves sometimes as a skeleton key for complex or unclear diagnostic scenarios (Verheul & Widiger, 2004), our main goal is to investigate how the variability within this category is reflected in terms of diagnostic accuracy, different development of the therapeutic and rehabilitative course, and of different outcomes at the end of the treatment.

**Methods:**

To reach this goal, we collected data on all patients referred with a PDNOS diagnosis and compared their treatment program. scenarios.

**Results:**

Our data showed how a PDNOS diagnosis hid in most cases complex personality disorders and comorbidities that reflected different specific difficulties and interventions during their treatment and, consequently, resulted in different outcomes.

**Conclusions:**

Our experience led us to give additional attention to referred PDNOS diagnosis and to observe how much a clear diagnostic picture of a patient is crucial to correctly plan a treatment program and adapt local service interventions both for personality disorder and comorbidity

